# Uracil/ftorafur/leucovorin combined with irinotecan (TEGAFIRI) or oxaliplatin (TEGAFOX) as first-line treatment for metastatic colorectal cancer patients: results of randomised phase II study

**DOI:** 10.1038/sj.bjc.6603493

**Published:** 2007-01-23

**Authors:** E Bajetta, M Di Bartolomeo, R Buzzoni, L Mariani, N Zilembo, E Ferrario, S Lo Vullo, E Aitini, L Isa, C Barone, S Jacobelli, E Recaldin, G Pinotti, A Iop

**Affiliations:** 1 Department of Medical Oncology, Unit 2, Istituto Nazionale per lo Studio e la Cura dei Tumori of Milano, Milano, Italy; 2 Statistics and Biometry Unit, Istituto Nazionale per lo Studio e la Cura dei Tumori of Milano, Milano, Italy; 3 Department of Medical Oncology and Hematology, Az. Osp. C. Poma, Mantova, Italy; 4 Department of Medical Oncology, Ospedale Serbelloni, Gorgonzola (MI), Italy; 5 Department of Medical Oncology, Policlinico ‘Sacro Cuore-A. Gemelli’, Roma, Italy; 6 Department of Medical Oncology, Università ‘G. D'Annunzio’, Chieti Scalo (CH), Italy; 7 Department of Medical Oncology, Ospedale ‘Sacro Cuore- Don Calabria’, Negrar (VR), Italy; 8 Department of Medical Oncology, Ospedale di Circolo Fond. Macchi, Varese, Italy; 9 Department of Medical Oncology, Ospedale Civile, Latisana (UD), Italy

**Keywords:** UFT, irinotecan, oxaliplatin, metastatic colorectal cancer

## Abstract

This randomised phase II study evaluates the safety and efficacy profile of uracil/tegafur/leucovorin combined with irinotecan (TEGAFIRI) or with oxaliplatin (TEGAFOX). One hundred and forty-three patients with measurable, non-resectable metastatic colorectal cancer were randomised in a multicentre study to receive TEGAFIRI (UFT 250 mg m^−2^ day days 1–14, LV 90 mg day days 1–14, irinotecan 240 mg m^−2^ day 1; q21) or TEGAFOX (UFT 250 mg m^−2^ day days 1–14, LV 90 mg day days 1–14, oxaliplatin 120 mg m^−2^ day 1; q21). Among 143 randomised patients, 141 were analysed (68 received TEGAFIRI and 73 TEGAFOX). The main characteristics of the two arms were well balanced. The most common grade 3–4 treatment-related adverse events were neutropenia (13% of cases with TEGAFIRI; 1% in the TEGAFOX group). Diarrhoea was prevalent in the TEGAFIRI arm (16%) *vs* TEGAFOX (4%). Six complete remission (CR) and 19 partial remission (PR) were recorded in the TEGAFIRI arm (odds ratio (OR): 41.7; 95% confidence limit (CL), 29.1–55.1%), and six CR and 22 PR were recorded in the TEGAFOX group, (OR: 38.9; 95% CL, 27.6–51.1). At a median time follow-up of 17 months (intequartile (IQ) range 12–23), a median survival probability of 20 and 19 months was obtained in the TEGAFIRI and TEGAFOX groups, respectively. Median time to progression was 8 months for both groups. TEGAFIRI and TEGAFOX are both effective and tolerable first-line therapies in MCRC patients. The employment of UFT/LV given in doublet combination is interesting and the presented data appear comparable to equivalent infusion regimens described in the literature. The safety profile of the two combinations also allows an evaluation with other biological agents such as monoclonal antibodies.

Up until the mid-1990s, best supportive care was still a valid treatment option in the treatment of advanced metastatic colorectal cancer and 5-fluorouracil (5-FU) represented the mainstay chemotherapy. In the last decade, irinotecan (CPT-11), oral fluoropyrimidines, oxaliplatin (L-OHP) and monoclonal antibodies have been added to the armamentarium of treatment options. This, of course, presents a challenge. Fluorouracil infusion in association with CPT-11 or L-OHP has shown a good activity and tolerability profile in metastatic colorectal cancer; therefore, FOLFIRI or FOLFOX are now considered the first-line options (de Gramont *et al*, 2000; Douillard *et al*, 2000; Saltz *et al*, 2000; Mayer, 2004). Many randomised studies have proved the equivalence in terms of activity or overall survival between FOLFOX and FOLFIRI regimens with a slight difference in the safety profile. FOLFOX is characterised by neutropenia (G3-4: 41%), neurotoxicity (G3-4: 18%) and diarrhoea (G3-4: 12%), whereas FOLFIRI by neutropenia (G3-4: 46%) and diarrhoea (G3-4: 14%) (Goldberg *et al*, 2004; Tournigand *et al*, 2004; Colucci *et al*, 2005).

Uracil/ftorafur is an oral third-generation fluoropyrimidine and was synthesised by Hiller in 1967 (Milano *et al*, 2004). This drug consists of two main compounds: tegafur, which is a pro-agent of 5-FU, and uracil, which is a simple competitive inhibitor of dihydropyrimidine dehydrogenase (DPD) activity. After gastrointestinal absorption, tegafur is converted to 5-FU by the liver microsomal system, mainly by the *P*-450 cytochrome. In colorectal cancer patients, the concentrations of 5-FU in serum, tumour and normal mucosa at various intervals after the final dose of UFT have been examined (Ho *et al*, 1998). Whereas the serum 5-FU concentration decreased to very low levels by 24 h following the UFT dose, the intratumoral 5-FU concentration decresed to only about half, and drug levels in normal mucosa were maintained at least 48 h after the final dose (Sadahiro *et al*, 2001). However 5-FU concentrations in the normal mucosa were approximately one-third of those measured in tumour tissue. Three phase III studies have been conducted to compare the efficacy and toxicity of UFT and bolus 5-FU, both modulated by leucovorin (LV) (Carmichael *et al*, 2002; Douillard *et al*, 2002; Lembersky *et al*, 2006). The results documented that they are equivalent in efficacy and that UFT/LV has a more favourable toxicity profile, with less neutropenia, diarrhoea, nausea, vomiting and mucositis. Given the *in vitro* synergy between L-OHP and UFT in the HT29 cell xenograft model, and between CPT-11 and 5-FU, some phase I studies have been conducted to define the recommended dose (Louvet *et al*, 2000; Prince and Hill, 2000; Alonso *et al*, 2001). Therefore, in the light of the above, it is to be expected that the combination of UFT/LV with CPT-11 (TEGAFIRI) or L-OHP (TEGAFOX) will be at least as effective as the corresponding infusion regimen.

The aims of this multicentre randomised non-comparative phase II study are to evaluate the safety profile of TEGAFIRI or TEGAFOX as first-line treatment and to determine the therapeutic efficacy in terms of response rate, duration of response, time to progression and overall survival.

## MATERIALS AND METHODS

### Patient eligibility

Patients with metastatic colorectal cancer, previously untreated by chemotherapy for advanced disease, were eligible for this study. Adjuvant chemotherapy, if administered, must have been completed at least 6 months before enrolment in the study. Histological confirmation of colorectal adenocarcinoma and the presence of at least one unidimensionally measurable lesion was requested. The patients had to be 18–75 years of age, with ECOG performance status 0–2. Other eligibility criteria were: absolute neutrophil count ⩾2.0 × 10^9^ l^−1^ at least; platelets ⩾100 × 10^9^ l^−1^ or more, haemoglobin ⩾10 g dl^−1^; lactic deydrogenase (LDH) ⩽1500 U l^−1^; serum creatinine ⩽1.25 mg dl^−1^; serum bilirubin ⩽1 × upper normal limit (UNL), alanine aminotransferase (ALAT) or aspartate aminotransferase (ASAT) or alkaline phosphatase <2.5 × UNL. However, level of up to five times the UNL for alkaline phosphates, ALAT and ASAT were allowed in patients with liver metastases. The study was conducted according to the Good Clinical Practices and Declaration of Helsinki. Written informed consent was required. The study and all current amendments were approved by the Ethics Committees of all of the participating centres.

Contraceptive measures were required for patients with reproductive potential; patients were not included if they were pregnant or lactating, had a history of other cancer except cured basal cell carcinoma of skin and carcinoma *in situ* of the uterine cervix, or if they had not fully recovered from recent, major surgery (within 4 weeks). Other exclusion criteria were presence of organ allograft, CNS involvement or neurological or psychiatric disorders, that could interfere with treatment compliance, severe cardiac disease or a myocardial infarction within the previous 12 months, uncontrolled metabolic disorders or active serious infections, inflammatory bowel disease, bowel obstruction or history of chronic diarrhoea and malabsorption syndrome. Patients were also excluded from the study if they had active neuropathy or previous fluoropyrimidines toxicity.

### Study design and treatment

**Figure 1:**
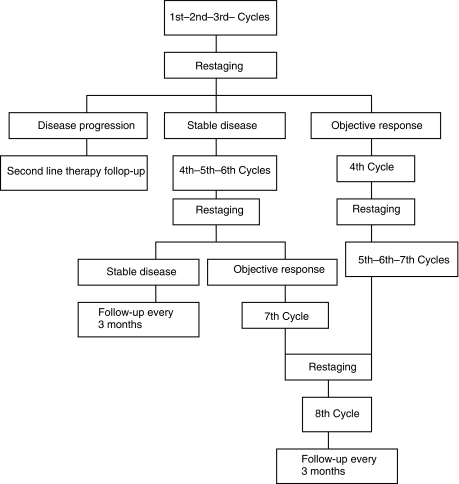
Study design.

This was an open-label, multicentre, randomised non-comparative Phase II study, conducted by the Italian Trials in Medical Oncology (ITMO) group and coordinated by Medical Oncology Unit 2. Patients who fulfilled the selection criteria were stratified by centre and by previous adjuvant chemotherapy and centrally randomised by the ITMO Scientific Office. TEGAFIRI consisted of UFT: 250 mg m^−2^ day and LV: 90 mg total dose daily, given for 14 days, combined with a 1-h infusion of CPT-11: 240 mg m^−2^ on day 1. TEGAFOX was administered as UFT: 250 mg m^−2^ day and LV: 90 mg total dose^−1^ daily, given for 14 days, combined with a 3-h infusion of L-OHP: 120 mg m^−2^ on day 1. The total daily UFT dose was divided to be given every 8 h; if the dose could not be equally divided, the greatest dose was administered in the morning. The treatment was given for a maximum of six cycles in presence of disease stabilisation or eight cycles in case of objective responses, as shown in Figure 1. The therapy was interrupted for unacceptable toxicity or consent withdrawal.

### Safety and efficacy analyses

Safety analyses included all patients who received at least one dose of the study medication. The analysis also included clinical assessments of adverse event reactions. Complete blood counts were obtained before every cycle and 21 days after the last day of chemotherapy.

The intensity of clinical adverse events was graded according to the NCI-CTC grading system (version 3.0). Adverse events not listed on the NCI-CTC grading system were graded as mild (grade 1), moderate (grade 2), severe (grade3) or life-threatening (grade 4). Hand–foot syndrome (palmar–plantar erythrodysesthesia-HFS) was classified as 3 grades: grade 1 (numbness, dysesthesia, painless swelling or erythema not disrupting normal activity); grade 2 (painful erythema with swelling affecting daily living activities); grade 3 (desquamation, ulceration, blistering or severe pain or any symptoms leading to an inability to work or perform daily living activities).

All cases who had received at least three cycles of study treatment and had at least one tumour assessment were considered evaluable for activity. Patients who failed to follow-up or who refused therapy were also included. Basal evaluation was performed within 28 days before starting treatment. Tumour dimensions that had a minimum size of at least one diameter of 10 mm were assessed using computerised tomography scans and magnetic resonance imaging. Figure 1 shows the treatment plan and the planned disease revaluation. All responses were confirmed and complete remission (CR), partial remission (PR), stable disease (SD) and progressive disease (PD) were defined according to the response definitions of the RECIST criteria (Therasse *et al*, 2000).

### Treatment modifications

In individual patients, treatment interruption or dose reduction was not indicated for reactions unlikely to become serious or life threatening, or for grade 1 toxicity. Treatment was interrupted in cases of grade 2 toxicity or worse, and it was resumed once the adverse event resolved or improved to grade 0 or 1.

For patients experiencing grade 3–4 neutropenia and/or thrombocytopenia, and/or febrile neutropenia, at the moment of recycle, the treatment was delayed for 1–2 weeks to allow for recovery, and then the dose reduced by 25% of the all study drugs. For patients who experienced a second occurrence of grade 3–4 toxicity, a further reduction by 50% was permitted. Treatment was definitely discontinued in cases of new occurrence of grade 3–4 toxicity.

In the presence of grade 2–4 diarrhoea, the UFT/LV administration was interrupted until recovery. The drug was then restarted with a reduction in dose by 25% in presence of grade 3–4 toxicity or with the second appearance of grade 2 diarrhoea. If grade ⩾2 HFS and/or mucositis occurred, the UFT administration was immediately stopped.

To ensure that the patient has been complying adequately with their medication regimen, at each visit the returned medication was checked and counted and the amount returned recorded in the drug dispensing log. If the patient stopped treatment, for any other reason other than side effects, for more than 1 week, he or she was withdrawn from the trial for non-compliance.

### Statistical analysis

The objective of the study was to evaluate separetely in each of the two study arms the safety and efficacy profiles of the two regimens in terms of the onset of grade 3–4 side effects (at least two sequential episodes of grade 3 or one episode of grade 4 neutropenia or diarrhoea) and the response rate (RR). The response rate was estimated as the fraction of evaluable patients who showed CR or PR; the corresponding 95% confidence limits were obtained by means of exact binomial calculation. The toxicity rate and corresponding confidence limits were calculated in the same manner. A sample of 60 subjects in each study arm had been planned following the Bayesian approach proposed by Thall *et al* (1995). This approach allowed early trial discontinuation in case of either insufficient antitumour activity or excessive treatment toxicity and was checked by simulation assuming an increase in the toxicity rate above 40% and/or a reduction of the response rate below 35%.

## RESULTS

Between July 2002 and November 2004, 143 patients were randomised by 14 Italian Institutions. Two patients were not analysed because they were never treated (rapidly progressive disease and ineligibility criteria); 68 patients were assigned to TEGAFIRI and 73 to TEGAFOX. Table 1 shows the main demographic and baseline characteristics that were comparable between treatment arms. Most patients had received no prior adjuvant therapy. Synchronous metastasis were documented in 72% of TEGAFIRI and in 67% of TEGAFOX cases. Twenty-one (31%) patients assigned to TEGAFIRI and 32 (44%) assigned to TEGAFOX had the liver as the only metastatic site. Baseline LDH values were elevated mostly in TEGAFIRI patients. However, elevations over 1.500 Ul^−1^ were not allowed by protocol criteria. CEA value was elevated in 56% of TEGAFIRI and in 53% of the TEGAFOX group.

## SAFETY ANALYSIS

All 141 patients who received at least one cycle of study medication were evaluable for safety. A total of 362 TEGAFIRI cycles and 411 TEGAFOX cycles were administered, with a median of six courses per patient (range 1–8) in both arms. Treatment was discontinued owing to toxicity in 11 TEGAFIRI (nine for gastrointestinal syndrome, and two for haematological side effects) and four TEGAFOX patients (three for allergic reaction and one for haematological side effect). One 60-day death was recorded in the TEGAFIRI arm, and no deaths were recorded in the TEGAFOX arm. Regarding dose reduction, TEGAFIRI cycles were administered with only a UFT reduction in 44 cycles (12%) and with a CPT-11 reduction in 11 (3%), whereas 37 (10%) cycles were administered with reduction in the dose of both drugs. TEGAFOX cycles were administered with only a reduced dose of UFT in 59 cycles (13%) and with only a reduced dose of L-OHP in 9 (2%), whereas 16 (4%) cycles were administered with both drugs at reduced doses. Most of these reductions consisted of 75% of the initial dose.

Table 2 shows the adverse events reported during the treatment with TEGAFIRI and TEGAFOX. The most common grade 3–4 treatment-related adverse events were neutropenia, which was reported in 13% of cases with TEGAFIRI and in 1% in the TEGAFOX group. Diarrhoea was prevalent in the TEGAFIRI arm (16%) *vs* TEGAFOX (4%). No HFS was reported in the TEGAFIRI group whereas grade 1–2 HFS was evident in 10% of the TEGAFOX group. Abdominal pain, allergic reactions, infection, liver toxicity and infection with fever were recorded as other toxicity. However, the overall incidence of any type grade 3–4 side effects was reported in 37–21% of TEGAFIRI and TEGAFOX patients, respectively.

Sequential grade 3–4 toxicity (as defined in the Materials and methods section) was recorded in four (5.9, 95% CL, from 1.6 to 14.4%) TEGAFIRI patients and three (4.1, 95% CL, from 0.9 to 11.5%) TEGAFOX patients.

## EFFICACY ANALYSIS

Eight patients in the TEGAFIRI group and one in the TEGAFOX group interrupted therapy soon after the first cycle, owing to side effects with no clinical benefit, and they were excluded. Thus, a total of 60 TEGAFIRI cases and 72 TEGAFOX patients were evaluable for efficacy analysis. Among these excluded cases, all except two were more than 65 years old. Table 3 shows study results on the best overall response rates. Six CR and 19 PR were recorded in the TEGAFIRI arm, for an overall response rate of 41.7% (95% CL, from 29.1 to 55.1%). In the TEGAFOX arm, six CR and 22 PR were recorded, corresponding to an overall response rate of 38.9% (95% CL, from 27.6 to 51.1).

The median duration of response was 6 (range: 3–15) for TEGAFIRI and 6 months (range: 3–23) for TEGAFOX group.

**Figure 2:**
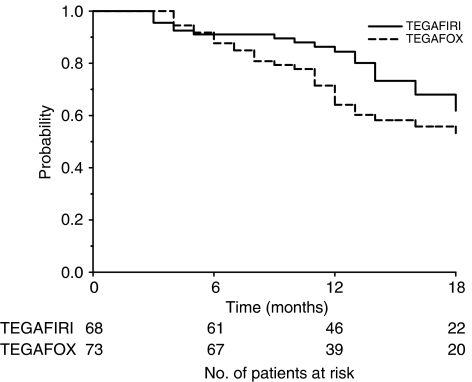
Overall survival by treatment.

**Figure 3:**
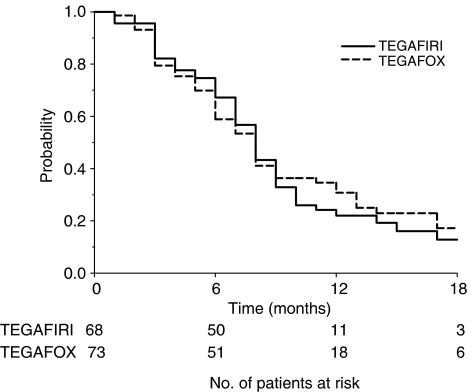
Time to progression by treatment in analysed patients.

After a median time follow-up of 17 months (IQ range 12–23), a median survival probability of 20 (IQ range 14–31) and 19 (IQ range: 11–29) months was obtained in the TEGAFIRI and TEGAFOX group, respectively. Median time to progression reported was 8 months (IQ range 5–11) for TEGAFIRI and 8 months (IQ range: 5–14) for TEGAFOX patients. The overall survival and time to progression are shown in Figures 2 and 3 respectively.

## DISCUSSION

The introduction in clinical practice of combination therapies such as FOLFIRI or FOLFOX has been an important development in colorectal cancer patient treatment. But, at the same time, it also creates some disadvantages such as the requirement of central venous catheters (CVC), infusion pumps or repeated intravenous administrations that are uncomfortable for the patients. Moreover, positioning of CVC could be complicated by pneumothorax, local infection, thrombosis and the frequent ambulatory visits that may have a negative impact on quality of life.

To decrease the level of these complications, new oral 5-FU prodrugs were introduced into the clinical practice. These agents such as UFT and capecitabine have demonstrated a relevant antitumour activity in preclinical trials and the pharmacokinetic profile of these drugs is equivalent to infusion 5-FU (Ishikawa *et al*, 1998). The role of oral fuoropyrimidines as a backbone of combination regimens with L-OHP and CPT-11 is an open question, although some phase II studies were carried out to evaluate the safety profile and activity of the capecitabine-based combinations (Bajetta *et al*, 2004; Reddy, 2005; Twelves *et al*, 2005). Whereas phase III trials comparing capecitabine-based and infusion 5-FU-based combination regimens are ongoing, there is no phase III trial evaluating the use of UFT/LV-based combinations.

This randomised phase II study evaluates whether the introduction of UFT, modulated by LV, in combination schemes determines a reduction of typical side effects with a satisfactory efficacy with respect to infusion regimens FOLFIRI and FOLFOX.

The data obtained documents an incidence of grade 3–4 diarrhoea (16%) and grade 3–4 neutropenia (13%) in the TEGAFIRI group and grade 3–4 neurotoxicity (6%), grade 3 diarrhoea (4%) and grade 3 neutropenia (1%) in the TEGAFOX patients. Moreover, during the treatment, two sequentially occurring grade 3–4 toxicities were reported in only 6% of TEGAFIRI cases and 4% of TEGAFOX patients. It means that a good safety profile has been obtained by a slight dose reduction (25%) of the two combinations.

Regarding any type toxicity, the TEGAFIRI regimen (grade 3–4: 37%) shows an increase in incidence with respect to the TEGAFOX regimen (grade 3–4: 21%). Twelve percent of TEGAFIRI patients and 1% of the TEGAFOX group stopped treatment for side effects soon after the first cycle; in the TEGAFIRI group, all cases were more than 65 years old. This means that the TEGAFOX regimen could be considered more suitable for older patients.

Other phase II trials have investigated the use of these two combinations. The toxicity profile observed with TEGAFOX and TEGAFIRI in the present study compares favourably with that reported in other phase II trials (Feliu *et al*, 2004, (Mendez *et al*, 2005; Bennouna *et al*, 2006). In particular, the rate of TEGAFOX grade 3–4 neurotoxicity was around 14–15% in Bennouna and Feliu studies as compared with 6% in our study. This probably correlated with the maximum number of administered cycles according to the study design reported in our study. Regarding TEGAFIRI, the toxicity rates of neutropenia and diarrhoea were comparable with those reported by Mendez.

However, these toxicity profiles correspond to those reported combining capecitabine and CPT11 or L-OHP with the exception of the greater rate of HFS that is reported in 20% of patients treated with capecitabine (Bajetta *et al*, 2004; Reddy, 2005; Twelves *et al*, 2005). This side effect has not been reported frequently with UFT.

The results we achieved with the employment of UFT modulated with LV, when combined to CPT11 or L-OHP show an efficacy in terms of response rate, time to progression and overall survival, which was at least comparable to respective infusion regimens. Moreover the efficacy, with overall response rates of 42% (in TEGAFIRI) and 39% (in TEGAFOX), a median survival rate of 20 and 19 months in the TEGAFIRI and TEGAFOX group, respectively, does not differ significantly from those reported in different phase II studies with capecitabine combintions (31–50%) (Bajetta *et al*, 2004; Reddy, 2005; Twelves *et al*, 2005).

Although we are waiting for the results of comparative phase III studies with the employment of capecitabine, the UFT/LV combinations studied can be considered a valid therapeutic option in first-line therapy. It may be warranted to evaluate these combinations with biological therapies such as bevacizumab or cetuximab.

## Figures and Tables

**Table 1:** Main basal characteristics

	**No. of pts (%)**	
	**TEGAFIRI (No. 68 pts)**	**TEGAFOX (No. 73 pts)**
*Age (years)*		
Median (range)	61 (36–74)	62 (23–73)
< 70	61 (89.7)	66 (90.4)
⩾70	7 (10.3)	7 (9.6)

M/F:	40 (58.8)/28 (41.1)	39 (53.4)/34 (46.5)

*PS (ECOG)*		
0–1	58 (85.3)–10 (14.7)	66 (90.4)–5 (6.9)
2	—	2 (2.7)

*Site of primary lesion*		
Colon dx	15 (22.1)	15 (20.6)
Colon sn	34 (50)	40 (54.7)
Rectum	19 (27.9)	18 (24.7)

*No. of metastatic sites*		
1	31 (45.6)	43 (58.9)
2	26 (38.2)	15 (20.5)
⩾3	11 (16.2)	15 (20.5)

*Adjuvant chemotherapy*		
Yes	13 (19.1)	14 (19.2)
Altered LDH value	27 (40)	15 (20)

Abbreviations: ECOG=Eastern Cooperative Oncology Group; LDH, lactic deydrogenase; pts, patients; PS.

**Table 2:** Frequency of patients reporting adverse events

	**% pts**							
	**TEGAFIRI (No. 68 pts)**				**TEGAFOX (No. 73 pts)**			
**Grade NCI CTC**	**G1**	**G2**	**G3**	**G4**	**G1**	**G2**	**G3**	**G4**
Diarrhoea	16.2	25.0	14.7	1.5	13.7	12.3	4.1	—
Nausea/emesis	29.4	26.5	11.8	—	19.2	31.5	2.7	—
Thrombocytop enia	—	—	—	1.5	5.5	4.1	1.4	—
Neurotoxicity	—	—	—	—	30.1	13.7	4.1	1.4
Mucositis	1.5	—	1.5	—	4.1	4.1	—	—
Asthenia	10.3	2.9	1.5	—	2.7	8.2	4.1	—
Alopecia	4.4	—	4.4	1.5	—	—	—	—
HFS	—	—	—	—	5.5	4.1	—	—
Leucopenia	1.5	4.4	8.8	1.5	4.1	2.7	1.4	—
Neutropenia	4.4	2.9	11.8	1.5	1.4	8.2	1.4	—
Anaemia	8.8	2.9	2.9	—	4.1	1.4	—	—
Other	7.4	7.4	5.9	—	13.7	4.1	6.8	—

Any type	23.5	27.9	32.4	4.4	16.4	47.9	19.2	1.4

Abbreviations: HFS, hand–foot syndrome; NCI CTC=National Cancer Institute Common Toxicity Criteria; pts, patients.

**Table 3:** Efficacy analysis

**Best overall response**	**TEGAFIRI No. 60 pts (%)**	**TEGAFOX No. 72 pts (%)**
Complete response	6 (10.0)	6 (8.3)
Partial response	19 (31.7)	22 (30.6)
Stable disease	23 (38.3)	25 (34.7)
Treatment failure	12 (20.0)	19 (26.4)

Objective response	25 (41.7) (95% CL, 29.1–55.1%)	28 (38.9) (95% CL, 27.6–51.%1)

Abbreviation: CL=confidence limit.
